# Automated Detection and Segmentation of Synaptic Contacts in Nearly Isotropic Serial Electron Microscopy Images

**DOI:** 10.1371/journal.pone.0024899

**Published:** 2011-10-21

**Authors:** Anna Kreshuk, Christoph N. Straehle, Christoph Sommer, Ullrich Koethe, Marco Cantoni, Graham Knott, Fred A. Hamprecht

**Affiliations:** 1 Interdisciplinary Center for Scientific Computing, University of Heidelberg, Heidelberg, Germany; 2 École Polytechnique Fédérale de Lausanne, Lausanne, Switzerland; Dalhousie University, Canada

## Abstract

We describe a protocol for fully automated detection and segmentation of asymmetric, presumed excitatory, synapses in serial electron microscopy images of the adult mammalian cerebral cortex, taken with the focused ion beam, scanning electron microscope (FIB/SEM). The procedure is based on interactive machine learning and only requires a few labeled synapses for training. The statistical learning is performed on geometrical features of 3D neighborhoods of each voxel and can fully exploit the high z-resolution of the data. On a quantitative validation dataset of 111 synapses in 409 images of 1948×1342 pixels with manual annotations by three independent experts the error rate of the algorithm was found to be comparable to that of the experts (0.92 recall at 0.89 precision). Our software offers a convenient interface for labeling the training data and the possibility to visualize and proofread the results in 3D. The source code, the test dataset and the ground truth annotation are freely available on the website http://www.ilastik.org/synapse-detection.

## Introduction

The chemical synapse is the predominant means by which information is transferred and stored in the central nervous system. Analysis of synapse size, shape and distribution contributes essential information to the understanding of neural circuitry, its function and its plasticity.

Despite the advances in light microscopy, detailed structural analysis of synapses is still only possible with electron microscopy. With serial section transmission electron microscopy (ssTEM), synaptic density can be estimated by manually counting synapses within a large volume, or by stereological extrapolation from paired 2D images [1]–[4]. However, using fairly thick 2D slices severely impedes detection of synapses in cases when the synaptic cleft is oriented at a low angle with respect to the plane of imaging [5].

The recent introduction of focused ion beam/scanning electron microscopy (FIB/SEM)[6] with isotropic resolution approaching 5 nm has now opened the door to a direct detection and segmentation of all synapses in large volumes of tissue, without the need to resort to extrapolation from paired slices. When searching for synapses, the human observer is not limited to the imaging plane projections of the volume, but can also explore the planes orthogonal to it. A protocol for manual synapse detection in FIB/SEM data has recently been proposed in [7]. Still, even for the best quality EM images, manual detection of synapses remains a difficult, error-prone and time-consuming task, which calls for automated protocols to overcome the tedium of manual analysis.

To detect synapses in EM images, human experts follow a set of morphological criteria: the presence of the pre- or post-synaptic densities, a visible synaptic cleft and a nearby cluster of at least two vesicles. If an automated protocol was to be based on these criteria directly, it would require a segmentation of the entire volume to find the membrane apposition sites and a full segmentation of ultra-cellular structures to detect vesicles. Although the problem of automated segmentation of neural tissue has advanced significantly in recent years, it is not yet fully solved [8], [9]. Also, automated segmentation of vesicles is nontrivial, especially at lower resolution, and has not received much attention in the literature. Rather than explicitly implementing the currently used criteria, machine learning allows to imitate the overall decisions of a human. The prediction rules are learned automatically from examples, provided in the form of annotated images (the training dataset). A meaningful measure of success is how well the automated predictions on a separate test set agree with those of the human.

Our contribution proposes an automated approach of this type and shows, through quantitative evaluation on a set of 111 synapses, that state-of-the-art machine learning methods can now achieve detection rates comparable to those of humans for asymmetric synapses in FIB/SEM data. Even though our approach does not explicitly implement the morphological criteria listed above, it finds enough evidence in the geometric features, extracted from a local neighborhood of each voxel, to mimic the decisions of the human expert.

In the field of neuroscience, recent influential work along these lines has focused on tracing and segmentation of neurons ([10]–[18]) or automated segmentation of ultracellular structures ([19], [20]). In [9], automated synapse detection has been proposed in the course of a large-scale semi-automated volume reconstruction effort. However, this approach relies on correct partitioning of the entire volume into cells, which is still impossible by fully automated means. Finally, automated methods for synapses detection have already been proposed for fluorescence light microscopy [21], [22]. Since these rely on fluorescent pre-labeling of all synapses, they are not applicable to EM images.

On the conceptual side, we rely on machine learning methods that are currently transforming all of image analysis. On the software side, we build on ilastik [23] and on our previous work, briefly described in [24]. ilastik (www.ilastik.org) is a freely available interactive learning and segmentation toolkit, which relies on a rich family of generic (nonlinear) image features and a robust nonlinear classifier [25] to estimate the probability of belonging to a synapse for each individual voxel. The training of the classifier by means of a pointing device (mouse or tablet pen) is fully interactive in the sense that a real-time display of the current predictions allows the user to iteratively provide more labels and hence improve the classifier performance. Once the classifier has been trained on a tiny subset of data, it can automatically classify all voxels in the volume as synapse or non-synapse. Then, all connected components of adjacent voxels with a sufficiently high probability of belonging to a synapse are aggregated into synapse candidates. Finally, a deterministic post-processing step rejects synapse candidates with implausible sizes. We provide a software bundle comprising a simple and intuitive graphical user interface for annotation, the machine learning algorithms and 3D visualization.

## Results

The quantitative validation of the automated synapse detection procedure, as well as the evaluation of the human experts' error rate, was carried out on a test dataset of 111 asymmetric, presumed glutamatergic, synapses (see Materials and Methods section for details on data acquisition and gold standard generation).

For the evaluation of the error rate, a synapse candidate was considered to be a false positive, if its “ball” label from the human expert or its shape segmented by ilastik did not overlap with any ball in the gold standard dataset. If such an overlap was found, the corresponding gold standard ball was removed from the set of possible matches. Conversely, a false negative detection was counted, if a ball from the gold standard did not overlap with any of the synapse candidates; if such an overlap was found the corresponding synapse candidate was removed from the set of possible matches. Human errors were additionally reverified manually, to avoid assigning a detection error in case of a geometric disagreement between labelers, i.e. when two labelers labeled the same synapse at positions so far from each other, that their “ball” labels did not overlap.

### Human experts

The expert which only had 4 hours to label and verify the synapses, missed 11 synapses and found 20 false positives. The other two experts, unlimited in time, made 2 and 3 false negative and 7 and 8 false positive detections respectively. Most expert mistakes were made for different synapses, which is in line with the observations of [26] about attention-related errors of expert annotators of neurobiological images.

### Automated detection

**Figure 1:**
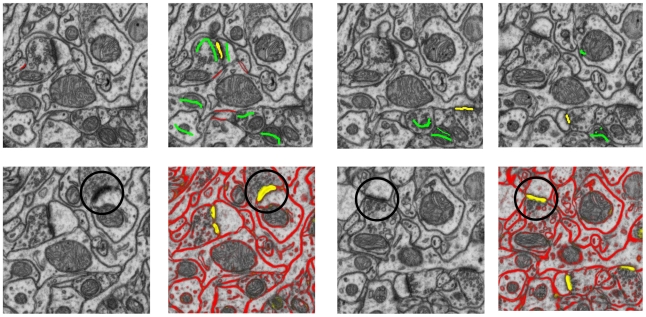
User labels and algorithm predictions. Top row: the complete set of user annotations for the first training set (20 brush strokes in total), with yellow labels for synapses, red for membranes, green for the rest. Bottom row: raw data and algorithm predictions on two other slices in the first training set. In black circles: some unlabeled synapses and their probability maps. The color intensity corresponds to the certainty in the prediction, predictions for green class are omitted for clarity.

**Figure 2:**
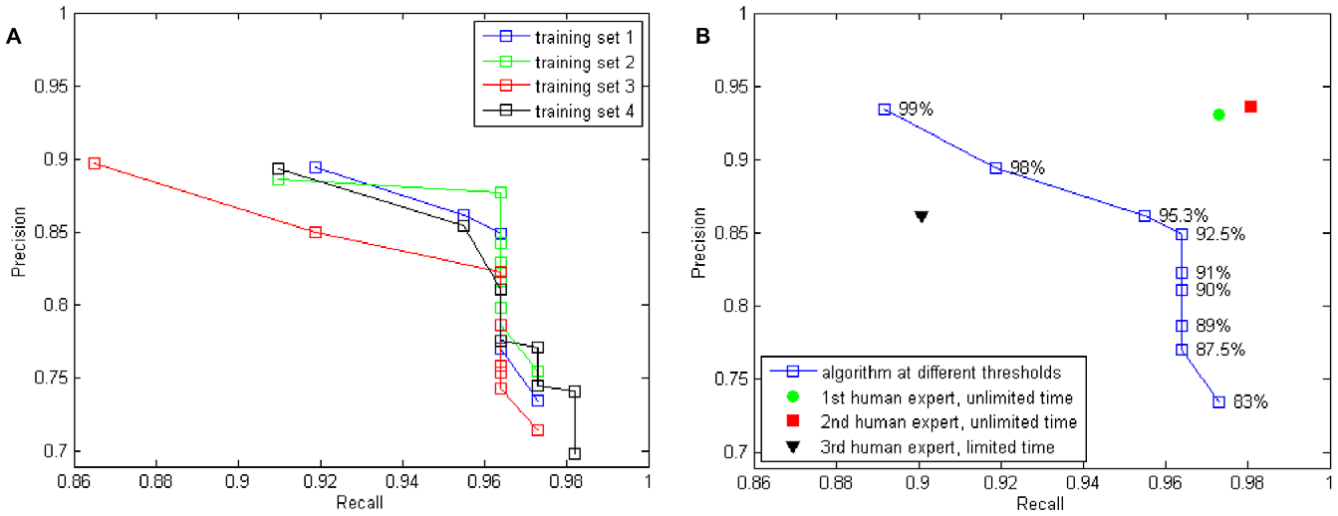
Precision and recall of the algorithm and the human experts. Recall was calculated as the (no. of true positives)/(no. of synapses in the ground truth), precision as the (no. of true positives)/(total no. of synapse candidates). **A**: Precision and recall of the algorithm results for the four different training sets. **B**: Precision and recall of the algorithm compared to the human experts with and without the time limit. The synapse probability threshold values are annotated next to the corresponding points of the curve.

To quantitatively assess the algorithm performance and its stability with regard to the training data, four training sets were created from images acquired in the same experiment, but not overlapping with the test set. The four training sets were located in different parts of the image stack and contained approximately the same number of voxel labels. For each training set, 2–3 synapses were labeled, and for each of those synapses it was sufficient to only label it in one of the slices. Adding more labels did not improve the classification performance, as long as the already labeled set represented the data well, which can be judged, for example, by looking at the current algorithm predictions for some non-labeled synapses (Fig. 1, bottom row). Although the software can discriminate an arbitrary number of categories, we found three-class labeling of synapses vs. membranes vs. the rest of the tissue to produce the best results. One can also use a binary setup with synapses vs. the rest, but then the labeler has to take extra care to annotate enough membrane voxels to obtain a representative sample of the background. Adding more classes, for example, for the mitochondria, did not help the classification. Our first training set is illustrated in Fig. 1 and a performance comparison for the different training sets is shown in Fig. 2A.

After training, the classifiers were applied to the test dataset, and thresholding with different sensitivity levels was applied to the resulting synapse probability maps. Precision and recall of the algorithm, depending on the threshold, are illustrated in Fig. 2 (using the training set from Fig. 1 for Fig. 2B). Recall was calculated as the (no. of true positives)/(no. of synapses in the ground truth), precision as the (no. of true positives)/(total no. of synapse candidates). The voxelwise threshold for the detection of synaptic cores was specified as the probability of the synapse class. For the training set from Fig. 1, the best algorithm performance was at the threshold of 98%, with recall of 0.92 and precision of 0.89. Overall, the algorithm performance is better than that of a human expert working with a four-hour time limit (0.9 recall and 0.86 precision), but worse than that of domain experts with unlimited time, who, in practice, worked on the problem on two consecutive days, though not all day long (recall of 0.97 and 0.98 and precision of 0.931 and 0.936). A comparable recall value for the algorithm (0.96) was achieved at precision of 0.85. Labeling the training set, computing its appearance features and training the classifier took approximately 15 minutes. Running the algorithm on the full test dataset took several hours, however, no user interaction was needed during this time.

**Figure 3:**
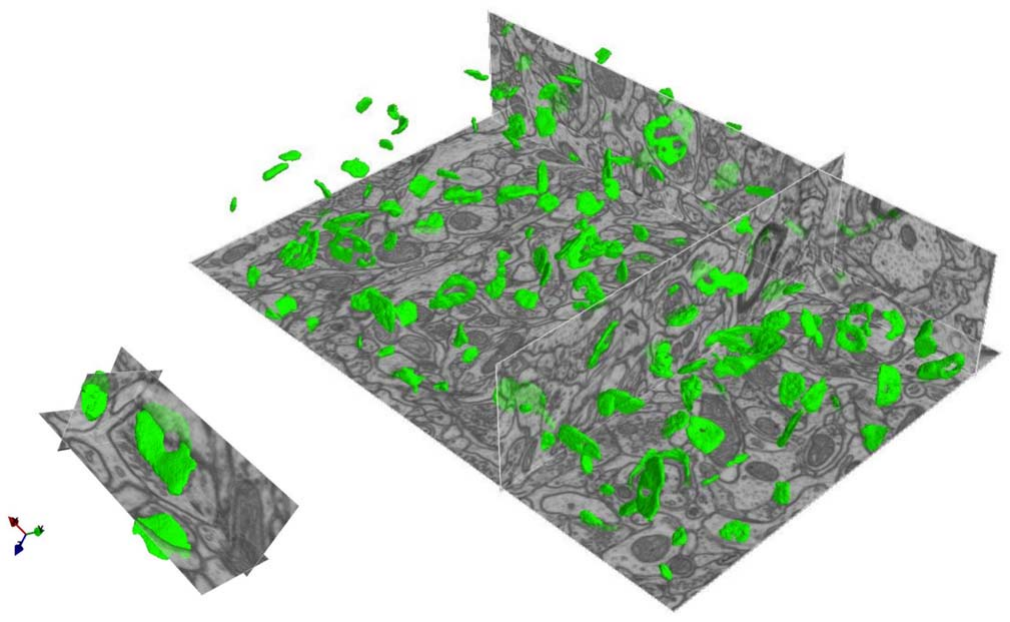
3D visualization of the results. Top: all synapses detected by the algorithm after training on the labels from Fig. 1. Bottom: a close-up view of three differently oriented synapses.

A 3D view of the synapses detected by the algorithm based on the training set from Fig. 1 (with probability ratio threshold of 92%) is illustrated in Fig. 3.

**Figure 4:**
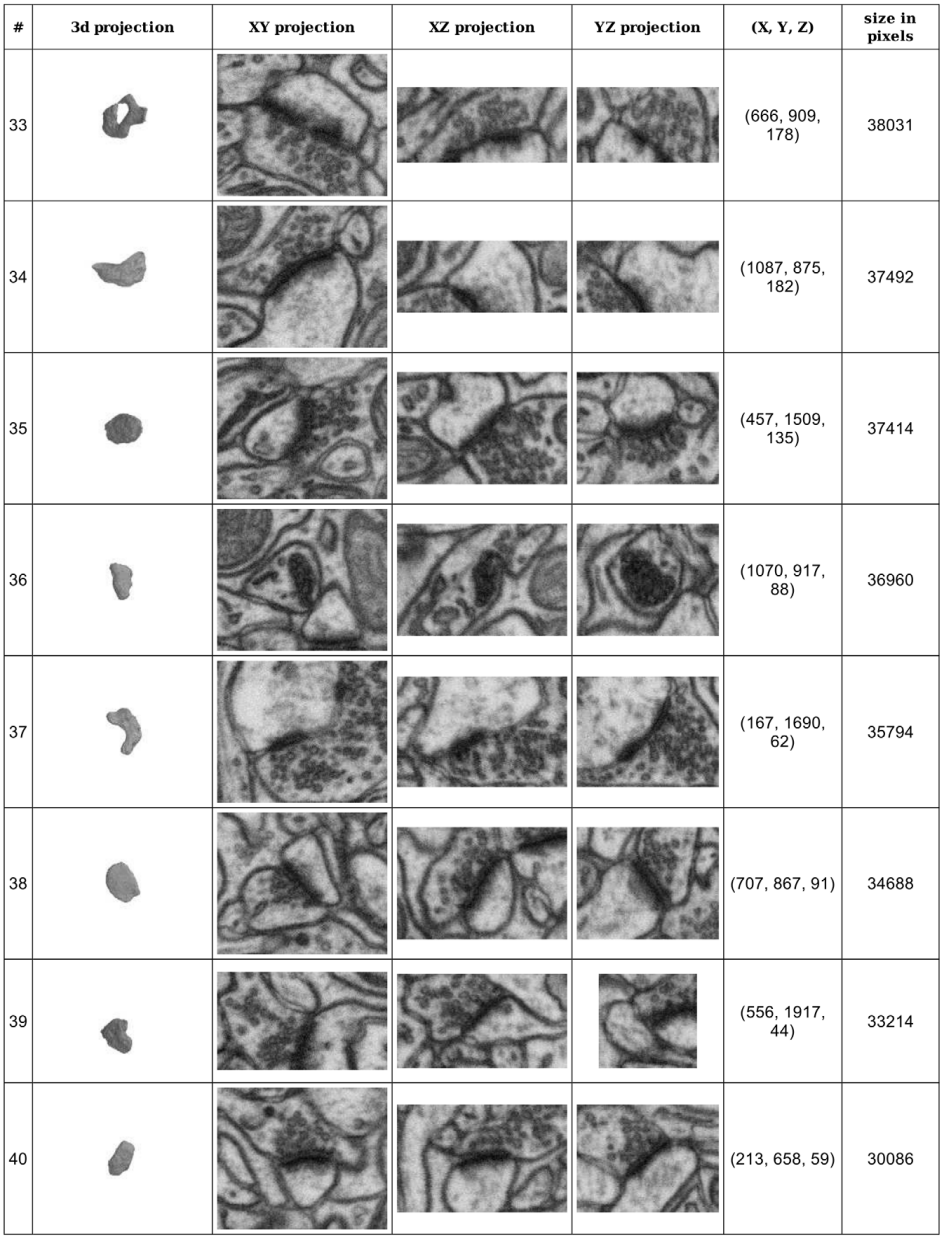
Synapse detection summary report. Part of the summary report produced by ilastik. The fourth detection from the top (no. 36) is a false positive, which can easily be filtered out by a human expert by looking at a larger context.

The human labelers only detected synapses and specified their approximate size by the ball labels, while the algorithm segmented synapses, i.e. listed every voxel belonging to a synapse candidate. Since the real synapses are not spherical, these human annotations can not serve as voxel-level gold standard. Consequently, the performance of the segmentation part of the algorithm was assessed qualitatively and found to be of sufficiently high quality for detailed analysis of synapse morphology, see Fig. 3 and Fig. 4.

## Discussion

**Figure 5:**
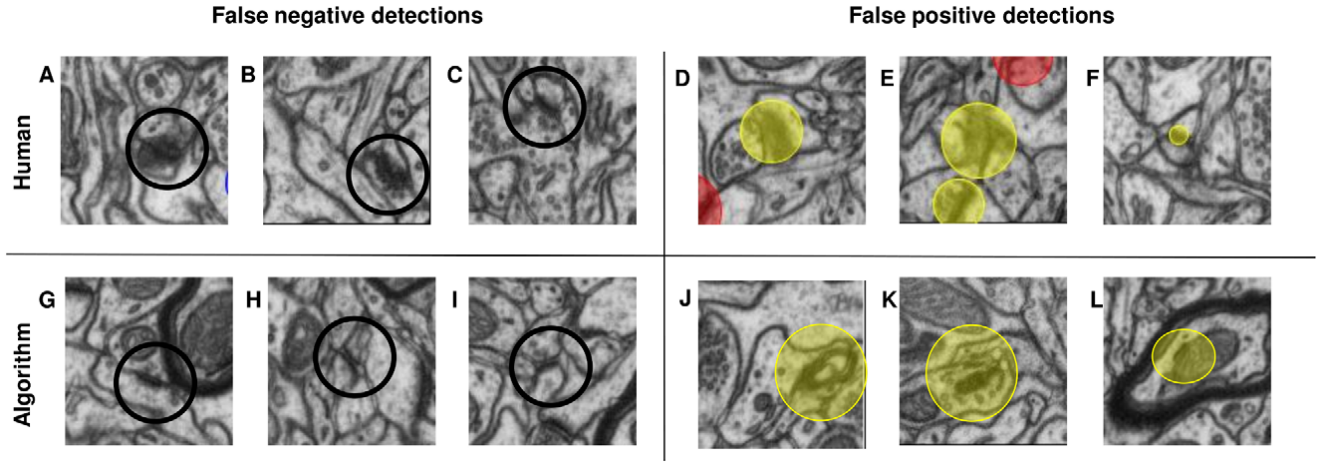
Error examples. **A, B, C:** false negative decisions of the human observers, **D, E, F:** false positive detections of the human observers, shown as yellow “ball” labels in the image center, **G, H, I:** false negative decisions of the algorithm, **J, K, L** false positive decisions of the algorithm.

The results show that with an adequate selection of appearance features, synapses are sufficiently different from other structures in neural tissue to allow for reliable automated detection in nearly isotropic FIB/SEM serial images. Fig. 5 illustrates typical false negative and false positive detections of the humans and of the algorithm, which have different causes. The false positives of the algorithm are mostly caused by myelinated membranes or very dark lines located near mitochondria (Fig. 5J, 5K, 5L). Similarly, most of the false negative detections also stem from synapses located very close to myelinated membranes. In the probability maps, they become connected to the large false positives caused by these membranes, and these large connected components are then filtered out based on the size criterion (Fig. 5G). Since ilastik provides a convenient summary report of all detected synapses (Fig. 4) and reduces the data from millions of voxels to just dozens of synapse candidates, the false positives for the entire stack can easily be discarded by a human in just a few minutes of additional proofreading.

For the human experts, while some synapses that were missed are accidental omissions, others serve as a good illustration of the advantages of truly 3D processing (Fig. 5A, 5B). These synapses are oriented at a low angle to the plane of imaging and do not strictly qualify as synapses according to the morphological criteria, since the synaptic cleft is not seen in the plane of imaging. Besides that, they are just hard to discern when viewing the data in native (x-y) projection only. Since the algorithm bases its decisions on geometric features computed in full 3D neighborhoods, it is not affected by synapse orientation.

As for any machine learning-based algorithm, the performance of ilastik depends significantly on how well the training dataset represents the true variability of the test data. Note also, that the images with the training labels must be large enough to allow for computation of all features from neighborhoods of the labeled voxels. The interactive learning interface of ilastik allows the user to immediately assess the algorithm performance on a subset of data and, if necessary, to modify the training labels or the threshold value. As shown in Fig. 2A, on our data the quality of the prediction was stable with respect to the exact choice of the training set.

We expect the proposed tool to be useful not only for synapse counting, synapse density estimation or estimation of synapse-to-neuron ratio, but also for the ongoing efforts in the reconstruction of neural circuits [8], [9], [26]–[29]. We are currently working on new machine learning methods which take more spatial context into account with the aim of solving the myelinated membranes problem and achieving reliable synapse segmentation also in image stacks with low z-resolution.

### Software and data availability

The software runs on Linux, MacOS and Windows. The binaries for the three platforms along with the installation instructions and documentation can be found at www.ilastik.org/synapse-detection, and the full source code is available in a github repository: www.github.com/Ilastik/ilastik. The test dataset, the gold standard set of synapse annotations and one of our training label sets can also be downloaded from the website. A small downsampled test dataset is also available as part of the supporting information (Dataset S1).

## Materials and Methods

### Data acquisition and generation of the gold standard

The test dataset consisted of 409 scanning electron micrographs from layer 2/3 of the adult rat somatosensory cortex. The tissue preparation methods followed the protocol previously described in [6] and were performed in accordance with the procedures approved by the Office Vétérinaire Cantonale Lausanne (license number 2106). Briefly, the brain of an adult rat was fixed by cardiac perfusion of 2.5% glutaradehyde, and 2% paraformaldehyde in phosphate buffer, it was then vibratome sectioned and slices from the somatosensory cortex were stained with buffered potassium ferrocyanide and osmium, followed by osmium, and then uranyl acetate. These stained sections were then dehydrated and embedded in Durcupan resin. The selected region was trimmed in an ultramicrotome and mounted onto an aluminium SEM stub for imaging in the FIB/SEM microscope (Zeiss NVision40), using a scanning electron beam at 1.3 kV with a current of 1 nAmp. Backscattered electrons were collected via the energy selective in-column detector (EsB) using a grid tension of 1.1 kV. The milling was achieved with a gallium ion source at 30 kV with a current of 700 pAmp. The acquired images were of 5 nm per pixel resolution with each image 1948×1342 pixels in size. The milling depth was measured at 9 nm per slice. Such high z-resolution allowed treating the data as one 3D volume of 1948×1342×409 voxels instead of a collection of 2D slices.

Synapses in the dataset were manually annotated by three independent human experts according to morphological criteria, including the presence of a pre- and post-synaptic density, as well as clustered vesicles close to the pre-synaptic membrane [30]. The human experts were researchers with experience in the analysis of electron micrographs of brain tissue and counting synapses in serial images. TrakEM2 plug-in of the FIJI framework [31] was used for the annotation. One of the experts only had four hours to label and verify the complete dataset, while the other two experts were not limited in time and took several hours longer. The annotation of each expert included positions and approximate size of detected synapses, denoted by “ball” labels from TrakEM2. Some examples of expert labels can be seen in Fig. 5D, 5E, 5F. Each expert first analyzed the dataset independently from the others and the resulting three sets of annotations were compared automatically to find all discrepancies. Since the automatic comparison procedure found differences between the expert annotations, these cases had to be re-examined jointly by all experts to establish a gold standard annotation. Synapses touching the left or top border of the image, as well as those touching the last slice of the stack, were excluded from the final count. For evaluation purposes, we also excluded synapses which had their center in the first slice of the stack, to avoid the border effects described in the next section. The resulting set of 111 synapses formed the gold standard and was used to estimate the error rates of both the original human annotations and the results obtained by the algorithm.

### Algorithm

The input data for the algorithm consists of scanning electron micrographs of neural tissue, provided as a pre-registered image stack, and user labels on a tiny subset of the data. The labeling can be very sparse, as shown in Fig. 1. The standard EM protocol used to prepare the brain tissue for imaging gives high contrast not only to synapses, but also to other cellular structures, such as mitochondria. As a consequence, the classification cannot simply be based on the raw intensity values of individual voxels. Instead, more informative features are required that also encode geometrical properties of 3D voxel neighborhoods. Different features represent different properties of these neighborhoods and should be selected so as to allow for an effective discrimination of the labeled classes. For example, as synapses are darker than intracellular space, the average intensity would serve as a good feature to distinguish these two, but would not help to separate synapses from membranes or mitochondria. Edge detectors respond strongly to both membranes and endoplasmic reticulum. Texture features respond to synapses, but also pick up thick mitochondrial membranes. Rather than devise decision rules by hand, we use statistical learning from a labeled training set to infer robust classification rules.

Since features have to be computed for every voxel, memory consumption has to be taken into account for large volumes. To allow running of the algorithm on a modern desktop PC rather than a high-end server without compromising classification accuracy, we performed selection of features, based on their Gini importance [25]. The final list of 38 features is provided in Table 1. Although the user is free to re-adjust the list and try out new feature combinations, we do not expect it to be necessary, except for the adjustment of the neighborhood sizes to the resolution of the data. Due to boundary effects in the feature computation, the performance of the algorithm can decrease for voxels very close to the limits of the dataset, such as the voxels of the first and last scan of the stack.

**Table 1:** Voxel features.

Feature	Sigmas	# of channels
Eigenvalues of the Hessian matrix	1, 1.6, 3.5, 5	3
Eigenvalues of the structure tensor	1, 1.6, 3.5, 5	3
Intensity of the Gaussian-smoothed image	0.7, 1, 1.6, 3.5, 5	1
Gradient magnitude of the Gaussian-smoothed image	1.6, 3.5, 5	1
Laplacian of the Gaussian-smoothed image	1.6, 3.5, 5	1
Difference of Gaussians	1.6, 3.5, 5	1
Total number of channels		38

Local neighborhood features, used for voxel classification. The “Sigmas” column shows the standard deviation of the Gaussians, used for smoothing the data. This parameter effectively determines the size of the necessary voxel neighborhood. For the eigenvalues of the structure tensor, the second scale parameter was set to sigma/2, for the difference of Gaussians the second Gaussian sigma was set to 0.66*sigma.

Based on the features and user labels, the Random Forest classifier [25] computes a probability map for each voxel, i.e. its probability of belonging to one of the classes defined in the training phase. Random Forest is a bagged ensemble of randomized decision trees that has only two parameters: the number of trees and the number of features considered at each split. Random Forest has been empirically shown to be fairly robust to their choice, and to provide very good results for a broad range of applications [32]–[35]. An example of Random Forest probability maps is shown in Fig. 1 (note the soft borders of the classes, which show that it's a probability estimate, not a hard segmentation).

The obtained probability maps are smoothed by convolution with a Gaussian with a standard deviation of 5 voxels to avoid local discontinuities caused by noisy voxel-wise predictions. Uncertain detections are then filtered out by considering only those clusters of voxels with synapse probability greater than a given threshold and with size of at least 1000 voxels. The lower limit for the size filter was computed as the approximate volume occupied by two vesicles at the given data resolution. The probability threshold can be interactively adjusted by the user. After thresholding, only the cores of synapses, i.e. areas of very high synapse probability, are left. These cores underestimate the real size of synapses, so to transition from detection to a proper segmentation we relax the synapse probability threshold to 0.5 for all voxels that are adjacent to synaptic cores.

### Software

The freely available ilastik toolkit [23] provides an intuitive interface for classification and segmentation of 2D and 3D data. In the interactive mode, it allows the user to immediately see the effect of newly added labels on the classifier's predictions, and therefore reduces the necessary labeling time. Once the classifier has been trained on a representative subset of the data, predictions on a very large dataset can be performed off-line in batch-processing mode.

Here we present and evaluate an extension of ilastik which includes interactively adjustable thresholding and finding of connected components, as well as a possibility to display the found objects in 3D with the help of the VTK toolkit [36]. A script for off-line thresholding and filtering is provided at [www.ilastik.org/synapse-detection]. Synapse detection results are stored in an hdf5-based ilastik project file and in an HTML summary report for convenient visualization and proofreading (Fig. 4). Integration of ilastik with the VTK visualization allows the user to jump from a 3D object directly to its position in the image stack.

## Supporting Information

Dataset S1 A small downsampled subvolume of the original data for trying out the interactive prediction.
(H5) Click here for additional data file.
